# Antibacterial Synthetic Peptides Derived from Bovine Lactoferricin Exhibit Cytotoxic Effect against MDA-MB-468 and MDA-MB-231 Breast Cancer Cell Lines

**DOI:** 10.3390/molecules22101641

**Published:** 2017-09-29

**Authors:** Yerly Vargas Casanova, Jorge Antonio Rodríguez Guerra, Yadi Adriana Umaña Pérez, Aura Lucía Leal Castro, Giovanni Almanzar Reina, Javier Eduardo García Castañeda, Zuly Jenny Rivera Monroy

**Affiliations:** 1 Biotechnology Institute, Universidad Nacional de Colombia, Carrera 45 No 26-85, 11321 Bogotá, Colombia; yvargasc@unal.edu.co; 2 Pharmacy Department, Universidad Nacional de Colombia, Carrera 45 No 26-85, Building 450, Office 213, 11321 Bogotá, Colombia; jorarodriguezg@unal.edu.co (J.A.R.G.); jaegarciac@unal.edu.co (J.E.G.C.); 3 Chemistry Department, Universidad Nacional de Colombia, Carrera 45 No 26-85, Building 450, Office 213, 11321 Bogotá, Colombia; jaumanap@unal.edu.co; 4 Medicine Faculty, Universidad Nacional de Colombia, Carrera 45 No 26-85, Building 450, Office 213, 11321 Bogotá, Colombia; allealc@unal.edu.co; 5 University Children’s Hospital, University of Wurzburg, 97080 Wurzburg, Germany; Almanzar_G@ukw.de

**Keywords:** lactoferricin B, *E. coli*, breast cancer, cytotoxic effect, antibacterial activity, synthetic peptides

## Abstract

Linear, dimeric, tetrameric, and cyclic peptides derived from lactoferricin B, containing the RRWQWR motif, were designed, synthesized, purified, and characterized using RP-HPLC chromatography and MALDI-TOF mass spectrometry. The antibacterial activity of the designed peptides against *E. coli* (ATCC 11775 and 25922) and their cytotoxic effect against MDA-MB-468 and MDA-MB-231 breast cancer cell lines were evaluated. Dimeric and tetrameric peptides showed higher antibacterial activity in both bacteria strains than linear peptides. The dimeric peptide (RRWQWR)_2_K-Ahx exhibited the highest antibacterial activity against the tested bacterial strains. Furthermore, the peptides with high antibacterial activity exhibited significant cytotoxic effect against the tested breast cancer cell lines. This cytotoxic effect was fast and dependent on the peptide concentration. The tetrameric molecule containing RRWQWR motif has an optimal cytotoxic effect at a concentration of 22 µM. The evaluated dimeric and tetrameric peptides could be considered as candidates for developing new therapeutic agents against breast cancer. Polyvalence of linear sequences could be considered as a novel and versatile strategy for obtaining molecules with high anticancer activity.

## 1. Introduction

According to the World Health Organization (WHO), breast cancer is the most frequent cancer, impacting over 1.5 million women each year, and also it causes the greatest number of cancer-related deaths among women. In 2015, 570,000 women died from breast cancer, which is approximately 15% of all female deaths causes by cancer [1]. The treatment of the disease consists of surgery, chemotherapy, radiotherapy, and drugs. However, the side effects are harmful, decreasing the quality of life of the patient. These treatments prolong the survival time, but recurrence of the disease is frequent. Chemotherapy causes several adverse effects, such as neutropenia, nausea and vomiting, amenorrhea, alopecia, neurological toxicity, weight gain, secondary leukemia, and cardiotoxicity [2,3]. Hormone therapy also causes adverse effects, such as hot flashes, musculoskeletal pain, fatigue, mood disturbances, nausea, vomiting, and fractures, among others [4].

Antimicrobial peptides (AMPs) exhibit a cytotoxic effect on cancer cell lines and antimicrobial activity because of the electrostatic interaction between the amino acid side chain and the negative charge of the membrane surface [5,6]. AMPs are able to discriminate between neoplastic and non-neoplastic cells, interacting specifically with negatively-charged membrane components such as phosphatidylserine, sialic acid, or heparan sulfate, which differ between cancer and non-cancer cells [7]. AMPs are considered to be a main source of molecules that are candidates for developing new drugs based on peptides. Bovine lactoferrin (BLF) is a milk protein with great biological activity. LF exhibits antimicrobial activity against pathogenic bacteria, fungi, parasites, and viruses. Furthermore, BLF inhibits colon, esophagus, lung, and bladder carcinogenesis in rats when administered orally in the post-initiation stage. BLF has the capacity to reduce the metastatic properties of both MDA-MB-231 and MCF-7 cell lines [8]. The bovine lactoferricin LfcinB: ^17^FKCRRWQWRMKKLGAPSITCVRRAF^41^ is a 25 amino acid-peptide belonging to the N-terminal region of BLF [9,10,11,12]. It has been suggested that LfcinB is responsible for the antimicrobial activity of BLF, and LfcinB exhibits greater antimicrobial and anticancerigenic activity than BLF itself [13,14,15]. LfcinB contains aromatic amino acids, such as tryptophan and phenylalanine, and basic residues (arginine and lysine) that confer amphipathic properties [9,16]. These positively-charged residues interact electrostatically with the negative charges of the bacterial cell wall lipopolysaccharide (LPS), allowing the peptide to approach the bacterial membrane [9,16]. Thereupon, hydrophobic residues interact with the membrane lipid bilayer, causing its disruption and cell lysis [9,16]. LfcinB exhibits a cytotoxic effect against human cancer cell lines of breast, gastric, and colorectal cancer, leukemia, fibrosarcomas, melanomas, and colon cancer [17,18,19,20,21]. Subcutaneous administration of LfcinB to mice inhibits lymphoma cell metastasis to the liver and lungs [17,22]. The minimal motif (RRWQWR) exhibited cytotoxic activity in leukemia cells, and the dead cells were related to both Cathepsin B and caspase activity [17,23,24].

In the present paper, linear, dimeric, and tetrameric peptides were synthesized via solid-phase peptide synthesis (SPPS) and purified and characterized using reverse phase high performance liquid chromatography (RP-HPLC) and matrix-assisted laser desorption/ionization-time of flight mass spectrometry (MALDI-TOF MS). Peptide antibacterial activity (against *E. coli* strains) and cytotoxicity against two breast cancer cell lines were evaluated. Our results indicate that peptides derived from LfcinB that exhibit antibacterial activity also exhibit a cytotoxic effect in breast cancer cell lines. Tetrameric and dimeric peptides containing the minimal motif have the greatest cytotoxic effect against both MDA-MB-468 and MDA-MB-231 breast cancer cell lines. This activity was fast and dependent on peptide concentration.

## 2. Results and Discussion

The aim of this study was to establish if the polyvalence or molecular restriction of LfcinB-derived peptides increases its antibacterial activity and/or cytotoxic effect in breast cancer cell lines. Therefore, linear, dimeric, tetrameric, and cyclic peptides containing sequences derived from LfcinB were designed and synthesized through SPPS, using the Fmoc/tBu strategy.

**Figure 1:**
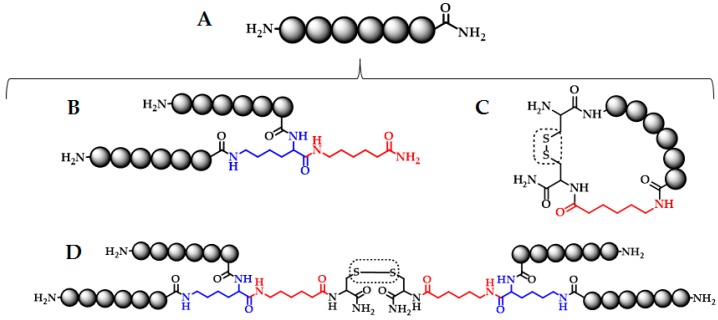
Designed and synthesized peptide families derived from LfcinB. Three families of peptides were obtained, each containing four members: a linear (**A**); a dimeric (**B**); a cyclic (**C**); and a tetrameric (**D**) peptide. As linear peptides, LfcinB (20–25), LfcinB (20–30), and [Ala^19^]-LfcinB (17–31) were used. The sequence is represented by black spheres; aminohexanoic residues (in red) and lysine residues (in blue) are shown.

Specifically, sequences LfcinB (20–25): ^20^RRWQWR^25^, LfcinB (20–30): ^20^RRWQWRMKKLG^30^, and [Ala^19^]-LfcinB (17–31): ^17^FKARRWQWRMKKLGA^31^ were selected. In order to obtain families of each sequence, all groups contained four members, specifically (i) a linear; (ii) a dimeric; (iii) a cyclic; and (iv) a tetrameric peptide, as it is shown in Figure 1.

**Figure 2:**
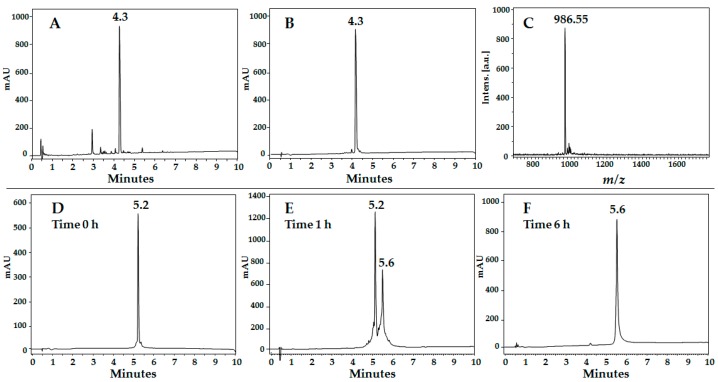
Reverse phase high performance liquid chromatography (RP-HPLC) analysis of LfcinB (20–25): crude (**A**) and purified (**B**) product. Purified LfcinB (20–25) matrix-assisted laser desorption/ionization-time of flight mass spectrometry (MALDI-TOF) mass spectrum (**C**). Tetramer LfcinB (20–25)_4_ formation by oxidation; RP-HPLC analysis of reaction mixture at different times: 0 h (**D**: purified dimer precursor, t_R_ 5.2 min); 1 h (**E**); and 6 h (**F**: final tetramer product, t_R_ 5.6 min).

Dimeric peptides (Figure 1B) were synthesized using the MAPs (multiple antigen peptides) methodology. The cyclic peptides (Figure 1C) were obtained by oxidation of cysteine residues located at the sequence C and N-terminal ends. Finally, the tetrameric peptides (Figure 1D) were obtained by the formation of an inter-disulfide bridge by the oxidation of purified dimeric precursor peptides (Table 1). All crude products were characterized using RP-HPLC and then purified by means of SPE. In all cases, the chromatographic profile of the purified products exhibited the main specie and purity was determined by RP-HPLC. MALDI-TOF-MS analysis showed that the synthesized peptides had the expected molecular weight (Table 1). As an example, Figure 2 shows the analytical results for peptide LfcinB (20–25); the chromatographic profile of crude product (Panel A) presents a main peak (t_R_: 4.3 min; purity: 40%). This product was purified and characterized, the RP-HPLC analysis shows a peak with the same retention time and a purity of 92% and its MALDI-TOF MS spectrum (Figure 2C) has a main signal at *m*/*z* 986,55 corresponding to [M + H]^+^. Oxidation reactions were monitored by RP-HPLC; Figure 2D presents the oxidation of a dimeric precursor, (RRWQWR)_2_K-Ahx-C, at reaction times of 0, 1, and 6 h, producing the tetramer (Lfcin B (20–25)_4_: (RRWQWR)_4_K_2_-Ahx_2_-C_2_).

The tetrameric peptide LfcinB (20–25)_4_, had shown antibacterial activity against (i) *E. coli* (ATCC 11775, MIC: 5.5 µM and ATCC 25922, MIC: 4.4 µM); (ii) *S. enteriditis* ATCC 13076 (MIC: 44 µM); and (iii) *S. aureus* ATCC 25923 (MIC: 25 µM). The dimeric peptide LfcinB (20–25)_2_ also showed antibacterial activity against the same bacterial strains with MIC 2.8 µM, 22.4 µM, 5.6 µM, respectively. Peptide LfcinB (20–25) had shown less antibacterial activity against these bacterial strains (MIC: 12.5 µM, >203 µM, 102 µM, respectively) [15,25,26]. These results showed that dimeric and tetrameric peptides containing the RRWQWR sequence have greater antibacterial activity against *E. coli* strains (ATCC 11775 and ATCC 25922) than the other tested bacterial strains. In a similar way, the tetramer LfcinB (20–25)_4_ exhibited greater cytotoxicity against oral squamous carcinoma cell lines than its linear peptide analogue, LfcinB (20–25) [27].

For this research, peptide activity was evaluated in two different models: (i) bacteria and (ii) breast cancer cell lines. First, the designed peptide families were tested against two *E. coli* strains (ATCC 11775 and 25922). It was found that dimeric and tetrameric peptides exhibit greater antibacterial activity against the evaluated strains than their linear counterpart sequences (Table 1), confirming that the polyvalence enhanced the antibacterial activity. This behavior is in accordance with the mechanism suggested for LfcinB, which involves the initial electrostatic interaction with the negative charges of the bacterial cell wall. It has been proposed that the increase of the positive charge of the molecules enables the interaction with the charged negative molecules of the bacterial surface [10,11,12,13,14]. It has been suggested that LfcinB peptides self-assemble, forming a polymeric structures as a requisite for the interaction with the bacterial surface [9,28]. On the other hand, cyclic peptides exhibit antibacterial activity similar to monomeric peptides, suggesting that molecular restriction of these amino acid sequences does not increase antibacterial activity in the evaluated strains. This indicates that the relevant properties for antibacterial activity are both positively charged and have an amphipathic sequence.

Many new therapies are currently being used for cancer treatment; among these new methods, chemotherapy based on antimicrobial peptides (AMPs) has been of great interest due to the unique advantages of this kind of molecule, such as low molecular weight, ability to specifically target tumor cells, and low toxicity in normal tissues [29]. For example, the cytotoxic effect of AMPs normally occurs at micromolar levels, and it is not accompanied by significant levels of hemolysis or toxicity to other mammalian cells. In most cases, the mechanisms underlying such activity involve disruption of mitochondrial or plasmatic membranes of the target tumor cells [5]. AMPs are considered to be promising molecules for developing new drugs for treating different cancer types. LfcinB is an AMP with potential for designing molecules with antibacterial and anticancer properties. In this context, it is important to identify short sequences derived from LfcinB with anticancerigenic activity, specifically against breast cancer.

**Figure 3:**
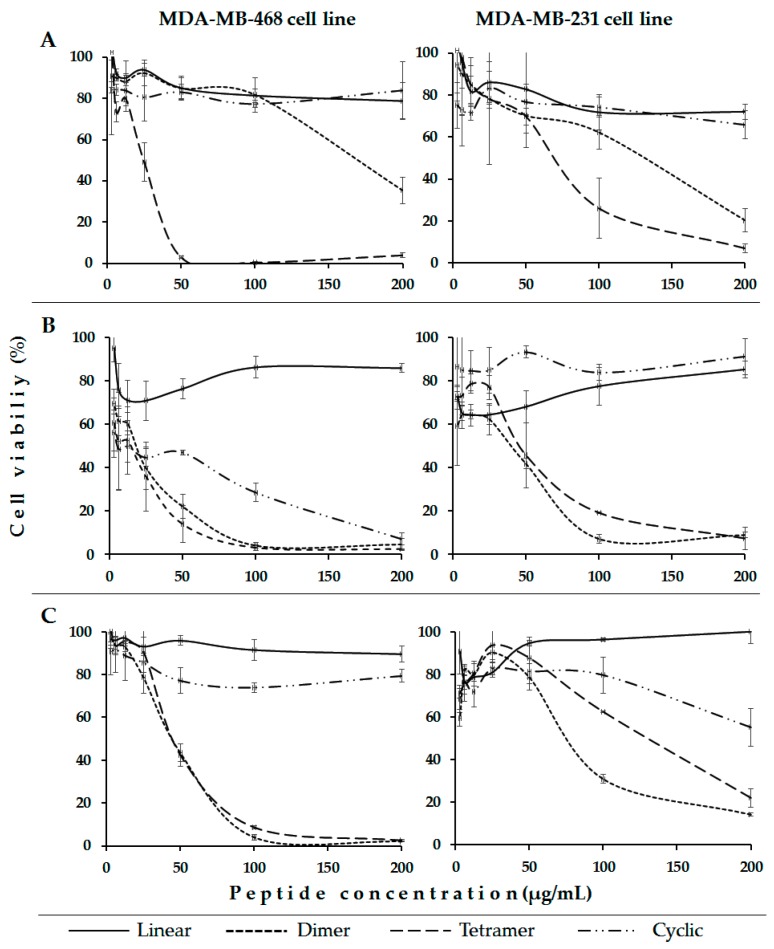
Cytotoxic effect of LfcinB-derived peptides against MDA-MB-468 (**left**) and MDA-MB-231 (**right**) breast cancer cell lines. Cytotoxic effect, after 2 h incubation time, of each designed peptide (Table 2) at different concentrations can be observed. LfcinB (20–25) family (**A**); LfcinB (20–30) family (**B**) and [Ala^19^]-LfcinB (17–31) family (**C**). The data are expressed as the mean ± S.D. (*n* = 3). Statistically significant differences were found in both cell lines at 100 µg/mL for: LfcinB(20–25)_4_ *cf* LfcinB (20–25), panel A; LfcinB(20–30)_4_ and LfcinB (20–30)_2_ *cf* LfcinB(20–30), panel B; and [Ala^19^]-LfcinB (17–31)_4_ and [Ala^19^]-LfcinB (17–31)_2_ *cf* [Ala^19^]-LfcinB (17–31), Panel C. (ANOVA, Post hoc Bonferroni, *p* < 0.05).

For the second part of the experiments, all members of each designed peptide family were tested against MDA-MB-468 and MDA-MB-231 breast cancer cell lines (Figure 3). It was found that the linear peptides LfcinB (20–25), LfcinB (20–30), and [Ala^19^]-LfcinB(17–31) exhibit a lower cytotoxic effect against both tested cell lines than the polyvalent peptides. When breast cancer cells lines were incubated with linear peptides or cyclic peptides (200 µg/mL), their viability was approximately 80%. Our results are consistent with other reports, which showed that neither T-leukemia nor MDA-MB-231 breast cancer cells were killed by free LfcinB (20–25); however, when this peptide was delivered (fusogenic liposome-mediated) into the cytosolic compartment, it caused extensive DNA fragmentation that was dependent on cathepsin B and caspase activation [17]. In a similar way, LfcinB (20–25) did not display any appreciable anticancer activity towards Jurkat cells, while the linear combination of hLF11 and LfcinB (20–25), which were joined as a single polypeptide chain by introducing either a Pro or a Gly-Gly spacer, considerably increased Jurkat-mediated cytotoxicity [24]. The linear peptides LFcinB (20–25) and LfcinB (20–30) exhibited a minimal cytotoxic effect (IC_50_ >500 µM) against human gastric cancer cell line AGS [17]. LfcinB and LfcinB (20–25) exhibited only weak cytotoxic activity against the adherent breast cancer cell line MDA-MB-231, even at the highest tested concentration (40 µM) [24].

It was found that the tetrameric peptide LfcinB (20–25)_4_ exhibited high cytotoxicity against both tested breast cancer cell lines, and that it is dependent on peptide concentration (Figure 3A). LfcinB (20–25)_4_ cytotoxic effects were greater in the MDA-MB-468 cell line than in the MDA-MB-231 cell line. In Table 2, the IC_50_ values indicate that peptides LfcinB (20–30)_2_, LfcinB (20–30)_4_, [Ala^19^]-LfcinB (17–31)_2_, and [Ala^19^]-LfcinB (17–31)_4_, showed also high cytotoxicity in both breast cancer cell lines, suggesting that the polyvalence could be relevant to the observed activity.

Specifically, the tetrameric peptide exhibits the maximum cytotoxic effect against MDA-MB-468 cell lines at a concentration of 11 µM (50 µg/mL), indicating that these cells are very sensitive to this molecule. The dimeric peptide LfcinB (20–25)_2_ (200 µg/mL) exhibited an intermediate cytotoxic effect in both tested cell lines. In a similar way, dimeric and tetrameric peptides containing the ^20^RRWQWRMKKLG^30^ sequence exhibited greater cytotoxic effect against both breast cancer cells lines than the linear sequence (Figure 3B). The dimer and tetramer exhibited the maximum cytotoxic effect at a concentration of 100 µg/mL, which corresponds to 30 µM and 15 µM, respectively, and their effect is constant at higher concentrations. In this family, the cyclic peptide showed a great cytotoxic effect against MDA-MB-468 cells, the cell viability being near to zero when the peptide concentration was 200 µg/mL (107 µM). The linear peptide exhibited a minimal cytotoxic effect in both breast cancer cell lines. For dimeric and tetrameric peptides containing the ^17^FKARRWQWRMKKLGA^31^ sequence, a greater cytotoxic effect against the breast cancer cell lines than their linear and cyclic analogues was also found (Figure 3C).

On the other hand, the dimeric peptides LfcinB (20–30)_2_ and [Ala^19^]-LfcinB (17–31)_2_ exhibited a greater cytotoxic effect than the dimeric peptide containing the minimal motif LfcinB (20–25)_2_, suggesting that the MKKLGA sequence enhanced the cytotoxic effect of those dimeric molecules. In all cases, tetrameric peptides exhibited a greater cytotoxic effect against MDA-MB-468 cell lines than that observed in MDA-MB-231 breast cancer cell lines.

**Figure 4:**
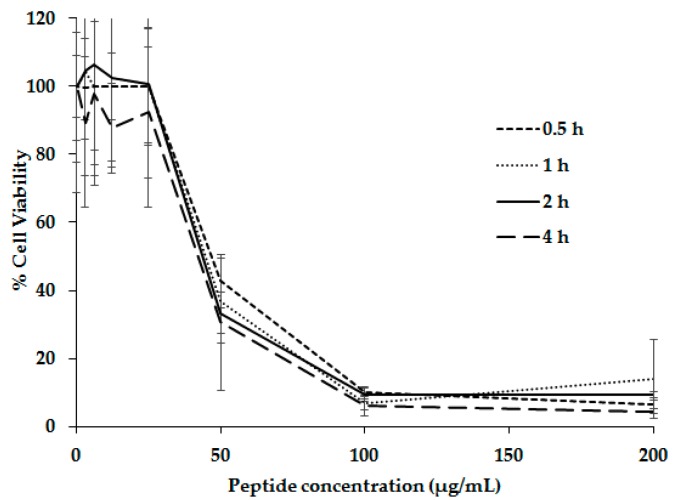
Cytotoxic effect of peptide LfcinB (20–25)_4_ against MDA-MB-468 cell line. Cells were treated with different peptide concentrations and different incubation times.

The cytotoxic effect of the tetrameric peptide LfcinB (20–25)_4_ against MDA-MB-468 breast cancer cell lines was tested at different incubation times, ranging from 30 to 240 min, with a peptide concentration of between 50 and 200 µg/mL (11–44 µM). It was found that at 30 min of treatment, the tetrameric peptide cytotoxic effect was significant, and after the first hour, cell viability was near 5% and was constant up to 4 h (Figure 4).

The results indicate that the cytotoxic effect is fast and independent of incubation time. It depends on peptide concentration, 100 µg/mL (22 µM) being the minimum concentration with maximum cytotoxic effect. This behavior was also observed for oral squamous-cell carcinoma (OSCC) cell lines, SCC15 and CAL27. When they were treated with this tetramer, its cytotoxic effect was significant after the first hour of treatment, and it was constant up to 24 h [27,30].

The peptides LfcinB (20–25)_2_ and LfcinB (20–25)_4_ exhibited a greater and faster cytotoxic effect on MDA-MB-231 cells than has been reported for LfcinB (after 18 h of incubation, 45% cell death) [17]. Similarly, dimeric and tetrameric peptides also exhibited a greater and faster cytotoxic effect than BLF and LfcinB in other cancer models: BLF and LfcinB exhibited cytotoxic activity and significantly stimulated the apoptosis of HT-29 cells. The maximum effects were observed at 12 h of treatment, and the optimal concentrations for BLF and LfcinB were 800 µg/mL and 400 µg/mL, respectively [18]. It has been reported that incubation (24 h) of human MDA-MB-435 breast carcinoma cells in the presence of LfcinB caused cell death by apoptosis [29].

**Figure 5:**
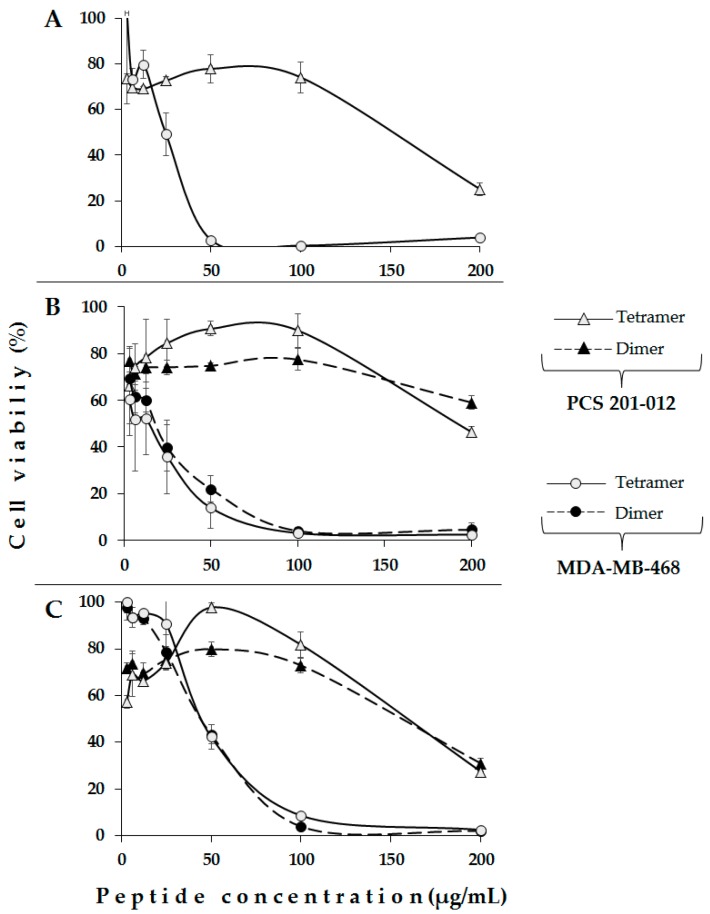
Comparative cytotoxic effect of LfcinB derived peptides against PCS 201-012 and MDA-MB-468 cell lines. (**A**) Peptide LfcinB (20–25)_4_; (**B**) LfcinB (20–30)_2_ and LfcinB (20–30)_4_ peptides and (**C**) [Ala^19^]-LfcinB (17–31)_2_ and [Ala^19^]-LfcinB (17–31)_4_ peptides.

Dimeric and tetrameric peptides that exhibited a cytotoxic effect against MDA-468 breast cancer cells, while exhibiting minimal cytotoxic effect in fibroblasts cells (PCS 201-012). The peptide LfcinB (20–25)_4_ exhibited great cytotoxic effects in MDA-468 cells at 50 µg/mL, while the cytotoxic effect was minimal in PCS-201-012 cells at the same concentration (Figure 5). Dimeric and tetrameric peptides containing ^17^FKARRWQWRMKKLGA^31^ and ^20^RRWQWRMKKLG^30^ sequences exhibited a similar behavior at 100 µg/mL (Figure 5). These results indicate that the cytotoxic effect of these peptides could be selective for breast cancer cells lines.

In Summary, the results indicate that polyvalence of linear sequences increases the antibacterial activity and cytotoxic effects against both oral and breast cancer cell lines. Dimeric and tetrameric peptides containing sequences shorter than LfcinB could be considered as candidates for developing new therapeutic agents against both breast and oral cancer.

It has been reported that both LFB and LfcinB has activity against different cancer types [11,24], we specifically had found that the LfcinB-derived tetramer has activity in oral cancer [30] and, herein, we show that LfcinB-derived peptides have selective cytotoxicity against breast cancer cells. These results are promissory and it is important to evaluate the cytotoxic effect in other breast cancer cell lines as well as in other normal epithelial cell lines (e.g., mammary, bladder, bronchial, corneal, prostate, and renal epithelial cell lines) to determine the spectrum of activity and the selectivity of these peptides. It is also important to establish if these peptides have antitumoral activity in animal model assays.

## 3. Materials and Methods

### 3.1. Reagents and Materials

Mueller-Hinton, Agar SPC, Mueller Hinton Broth (MHB), *E. coli* ATCC 11775 and *E. coli* ATCC 25,922 were obtained from ATCC (Manassas, VA, USA), Fetal Bovine Serum (FBS) was obtained of Gibco. *N*,*N*-diisopropylethylamine (DIPEA), triisopropylsilane (TIPS), 1,2-ethanedithiol (EDT), 4-methylpiperidine, pyridine, and ninhydrin were obtained from Sigma-Aldrich (St. Louis, MO, USA). Rink amide resin, Fmoc-amino acids, 6-chloro-1-hydroxy-benzotriazole (6-Cl-HOBt), and *N*,*N*-dicyclohexylcarbodiimide (DCC) were purchased from AAPPTec (Louisville, KY, USA). Methanol, diethyl ether, *N*,*N*-dimethylformamide (DMF), absolute ethanol, dichloromethane (DCM), acetonitrile (ACN), isopropylalcohol (IPA), and trifluoroacetic acid (TFA) were obtained from Honeywell-Burdick & Jackson (Muskegon, MI, USA). All reagents were used without further purification.

### 3.2. Solid Phase Peptide Synthesis

Peptides were synthesized using manual SPPS-Fmoc/tBu [25,31]. Briefly, Rink amide resin (0.46 meq/g) was used as solid support. (i) Fmoc group removal was carried out through treatment with 20% 4-methylpiperidine in DMF); (ii) For the coupling reaction, Fmoc-amino acids (0.21 mmol) were pre-activated with DCC/6-Cl-HOBt (0.20/0.21 mmol) in DMF at RT; (iii) Side-chain deprotection reactions and peptide separation from the resin were carried out with a cleavage cocktail containing TFA/water/TIPS/EDT (93/2/2.5/2.5 *v*/*v*/*v*); (iv) Crude peptides were precipitated by treatment with cool ethyl ether, dried at RT, and analyzed using RP-HPLC analytical chromatography.

### 3.3. LfcinB-Derived Peptide Characterization

#### 3.3.1. Reverse Phase HPLC

RP-HPLC analysis was performed on a Merck Chromolith^®^ C18 (50 mm × 4.6 mm) column using an Agilent 1200 liquid chromatograph (Omaha, NE, USA) with UV-Vis detector (210 nm). For peptide analysis (1.0 mg/mL crude or purified molecule), 10 μL samples were injected and a linear gradient was applied from 5% to 70% Solvent B (0.05% TFA in ACN) in Solvent A (0.05% TFA in water) for 11.5 min at a flow rate of 2.0 mL/min at room temperature.

#### 3.3.2. Peptide Purification

Molecules were purified using solid-phase extraction columns (SUPELCO LC-18 with 2.0 g resin). SPE columns were activated prior to use with 30 mL acetonitrile (containing 0.1% TFA) and equilibrated with 30 mL water (containing 0.1%TFA). Crude peptides were passed through the column, and a gradient was used for their elution. Collected fractions were analyzed using RP-HPLC (as described above). Fractions that contained pure products were lyophilized.

#### 3.3.3. MALDI-TOF MS

The purified peptides were analyzed by MALDI-TOF mass spectrometry. For sample preparation a solution of peptide (1 mg/mL) was mixed with the matrix (1.0 mg/mL of 2,5-dihydroxybenzoic acid, or sinapinic acid) in a relation of 2:18 (*v*/*v*) and 1 µL was seeded on the steel target. The experiment was performed on an Ultraflex III TOF-TOF mass spectrometer (Bruker Daltonics, Bremen, Germany) in reflectron mode, using an MTP384 polished steel target (Bruker Daltonics, Bremen, Germany), Laser: 500 shots and 25–30% power.

### 3.4. LfcinB-Derived Peptide Biological Activity

#### 3.4.1. Antibacterial Activity Assays

The minimal inhibitory concentration (MIC) was determined using a microdilution assay [26]. In brief, bacterial strains were incubated for 18 to 24 h at 37 °C in an Muller Hinton broth (MHB) until an optical density of 0.15 to 0.30 (620 nm) was obtained. 90 µL of MHB was mixed with 90 µL of peptide (440 µg/mL), and using a 96-well microtiter plate peptide, serial dilution (200, 100, 50, 25, 12.5, and 6.2 µg/mL) was performed. 10 µL of inoculum (2 × 10^6^ CFU/mL) was added to each well. Final volume in each well was 100 µL. Then they were incubated for 24 h at 37 °C, and the absorbance at 620 nm was measured using an Asys Expert Plus ELISA reader. For determining the minimum bactericidal concentration (MBC), a small sample was taken from each well using an inoculation loop, which was then spread on MHA plates and incubated overnight at 37 °C. MBC was considered to be the plate which exhibited no bacterial growth. Each of these tests was performed twice (*n* = 2).

#### 3.4.2. MTT Assay

Cytotoxicity assays were performed as previously described [32]. Briefly, breast cancer cell lines (100 µL; 2.5 × 10^3^ cells/well) were seeded in 96-well flat bottom tissue culture treated plates and were incubated at 37 °C in a 10% CO_2_ humidified atmosphere for 24 h, allowing for cell adhesion. Then the media was removed, then 100 µL of FBS (5%) and 100 µL of peptide were added. The peptide final concentration was ranging from 200 to 6.25 µg/mL and the final FBS concentration was 2.5%. Plates were incubated 2 h at 37 °C in a 10% CO_2_ humidified atmosphere (physiologic pH). Negative controls included medium and water. All controls were prepared in triplicate. Cell viability was determined using the MTT assay after 2 h [33]. For this, 10 µL of MTT solution (5 mg/mL) was added to each well, and the plates were incubated for 2 h at 37 °C. Formazan crystals were clarified by centrifugation, the supernatant was discarded, and the crystals were dissolved in DMSO (100 µL). Absorbance (570 nm) was registered on a Bio-Rad 680 microplate reader. (*n* = 3).

## Figures and Tables

**Table 1:** Analytical characterization summary of designed and synthesized LfcinB-derived peptides.

Synthetic Peptide Sequence	Analytical Characterization			
	RP-HPLC		MALDI-TOF MS	
	t_R_ (min)	Purity ^c^ (%)	Monoisotopic Mass (M)	Experimental *m*/*z*, [M + H]^+^
**^20^RRWQWR^25^**	4.3	92	985.54	986.55
(RRWQWR)_2_K-Ahx	5.1	86	2195.24	2198.46
(RRWQWR)_2_K-Ahx-C ^a^	5.2	86	2298.24	2300.93
C-RRWQWR-Ahx-C ^b^	4.4	85	1304.64	1306.08
**^20^RRWQWRMKKLG^30^**	4.7	95	1542.87	1544.53
(RRWQWRMKKLG)_2_K-Ahx	5.3	86	3309.91	3311.62
(RRWQWRMKKLG)_2_K-Ahx-C ^a^	5.4	90	3412.92	3415.07
C-RRWQWRMKKLG-Ahx-C ^b^	5.0	95	1861.98	1862.14
**^17^FKARRWQWRMKKLGA^31^**	4.9	95	1960.11	1961.99
(FKARRWQWRMKKLGA)_2_K-Ahx	5.5	88	4144.38	4146.73
(FKARRWQWRMKKLGA)_2_K-Ahx-C ^a^	5.5	85	4247.39	4255.51
C-FKARRWQWRMKKLGA-Ahx-C ^b^	5.2	92	2279.21	2279.93

^a^ Precursor of tetrameric peptide; ^b^ Precursor of cyclic peptide and ^c^ Peptide purity was calculated using the percentage of peak area at the chromatographic profile.

**Table 2:** LfcinB-derived peptides’ biological activity.

Peptide Code	Antibacterial Effect MIC/MBC µg/mL (µM)		Cytotoxic Effect IC_50_ (µM)	
	*E. coli* ATCC 11775	*E. coli* ATCC 25922	MDA-MB-468	MDA-MB-231
LfcinB (20–25)	200(203)/200(203)	200(203)/200(203)	>203	>203
LfcinB (20–25)_2_	50(22)/50(22)	12.5(6)/25(11)	>100	130
LfcinB (20–25)_4_	100(22)/200(44)	100(22)/100(22)	6	15
LfcinB (20–25)_cyc_	200(153)/200(153)	100(77)/>200(>153)	>200	>200
LfcinB (20–30)	200(130)/200(130)	200(130)/200(130)	>130	>130
LfcinB (20–30)_2_	200(60)/200(60)	100(30)/200(60)	5	14
LfcinB (20–30)_4_	200(15)/200(15)	200(15)/200(15)	2	6
LfcinB (20–30)_cyc_	200(107)/200(107)	200(107)/200(107)	>107	27
[Ala^19^]-LfcinB (17–31)	>200(>102)/>200(102)	200(102)/200(102)	>102	>102
[Ala^19^]-LfcinB (17–31)_2_	>200(>48)/>200(>48)	100(24)/100(24)	11	31
[Ala^19^]-LfcinB (17–31)_4_	ND	ND	5	9
[Ala^19^]-LfcinB (17–31)_cyc_	>200(>88)/>200(>88)	200(88)/200(88)	>88	>88
